# The Comet Toolbox: Improving robustness in network neuroscience through multiverse analysis

**DOI:** 10.1162/IMAG.a.1122

**Published:** 2026-02-10

**Authors:** Micha Burkhardt, Carsten Gießing

**Affiliations:** Department of Psychology, Psychological Methods and Statistics, Carl von Ossietzky University Oldenburg, Oldenburg, Germany; Department of Psychology, Biological Psychology, Carl von Ossietzky University Oldenburg, Oldenburg, Germany

**Keywords:** dynamic functional connectivity, multiverse analysis, graph analysis, fMRI, toolbox

## Abstract

In network neuroscience, a broad range of methods for estimating dynamic functional connectivity from fMRI data and subsequent analyses using network-based approaches have been introduced in recent years. However, in the absence of ground truths about the validity of analytical steps in capturing true brain dynamics, researchers are often faced with a multitude of arbitrary yet defensible choices, raising concerns about the robustness of results. Here, we aim to address this issue by implementing a comprehensive suite of dynamic functional connectivity methods in a unified Python software package, allowing for a diverse exploration of brain dynamics. Anchored in the framework of multiverse analysis, the present work introduces a workflow for systematically exploring different methodological choices. The developed toolbox includes a graphical user interface to enhance ease of use and accessibility for those who prefer to work outside a script-based pipeline. Comprehensive documentation and demo scripts are included to support adoption and usability. By promoting transparency and robustness, Comet aims to advance best practices in the study of brain dynamics.

## 1 Introduction

In recent years, the field of network neuroscience has witnessed a rapid expansion in methodological diversity, particularly in the estimation of (dynamic) functional connectivity, which often serves as the basis of subsequent network-based analyses. While this growth reflects clear methodological progress, it has also heightened uncertainty about how different analytical choices influence results. These concerns are not unique to neuroscience: Across disciplines such as psychology, economics, and genomics, it has become evident that flexibility in data processing and analysis can introduce substantial researcher degrees of freedom, inflating false-positive rates and shaping reported effects in systematic ways (Frias-Navarro et al., 2020; Ioannidis, 2005; Simmons et al., 2011). Despite increasing efforts to promote transparency, for example through open data, preregistration, or more rigorous reporting standards, a general replication crisis continues to challenge the scientific literature (Fanelli, 2018; Frias-Navarro et al., 2020).

Within neuroimaging, these concerns are amplified by the inherently noisy nature of functional MRI (fMRI) data and the many analytical decisions required throughout data processing, modeling, and statistical inference. Early studies already documented the substantial influence of such analytical variability. For example, Strother et al. (2002) systematically compared preprocessing and analysis strategies, demonstrating that different choices can lead to markedly different statistical outcomes. Subsequent efforts reinforced this conclusion: the FIAC experiment showed that expert teams applying distinct software suites and preprocessing pipelines to the same dataset produced notably divergent activation patterns (Poline et al., 2006). Botvinik-Nezer et al. (2020) recently extended these findings by showing that, across an even broader range of analysts and modeling choices, researchers analysing identical data can arrive at different conclusions depending on modeling assumptions, software, and particularly thresholding criteria. Similar concerns were also raised in the domain of functional connectomics. Recent work has demonstrated that variations in processing decisions can lead to substantial variability in the resulting static functional network topologies (Luppi et al., 2024). Kristanto et al. (2024) further detailed a number of heavily debated decision nodes throughout graph-based fMRI workflows, underscoring that analytic flexibility remains a central challenge in studying the network organization of the brain.

The aforementioned issues are further magnified in the context of dynamic functional connectivity (dFC), which aims to characterize time-varying patterns of functional interactions between brain regions as they reconfigure across cognitive states or in response to internal or external demands (Hutchison et al., 2013; Iraji et al., 2021). dFC has shown great promise in uncovering mechanisms underlying a range of neuropsychiatric and neurological disorders, including schizophrenia, Alzheimer’s disease, and mood disorders (Calhoun et al., 2014; Gonzalez-Castillo & Bandettini, 2018). A wide array of methods has been proposed for estimating dFC, including sliding-window correlation, co-activation patterns, time-varying graphical models, and Hidden Markov Models (Hutchison et al., 2013; Lurie et al., 2020; Preti et al., 2017). However, these methods often yield different results, and no consensus has emerged on best practices for estimating dFC (Thompson et al., 2018; Torabi et al., 2024). Moreover, test–retest studies reveal low-to-moderate reliability of dFC estimates, with intraclass correlation coefficients frequently ranging between 0.2 and 0.5 (Choe et al., 2017; Zhang et al., 2018). This combination of methodological diversity, inconsistent reliability, and absence of consensus highlights the need for systematic tools capable of evaluating how analytical choices influence conclusions drawn from dFC analyses.

Taken together, the concerns raised across neuroimaging reflect a broader recognition that analytical flexibility must be explicitly mapped and quantified to ensure robust and transparent scientific inference. Developments in the wider reproducibility literature have formalized these issues into explicit frameworks for characterizing analytical variability, among which multiverse analysis has emerged as a particularly influential approach (Steegen et al., 2016). Multiverse analysis makes explicit what is widely recognised by researchers, but frequently left implicit in publications: that many stages of a data processing and analysis workflow allow for multiple defensible alternatives, each of which can systematically influence research outcomes. Rather than relying on a single analytical pipeline, the multiverse approach systematically evaluates all defensible combinations of analytical decisions (spanning data preprocessing, modeling, and statistical inference) to examine how conclusions vary across the full decision space. Each combination of decisions constitutes a distinct “universe”, and comparing these universes allows researchers to quantify the robustness, sensitivity, and dependence of their findings on specific methodological choices (Del Giudice & Gangestad, 2021; Simonsohn et al., 2020).

Importantly, the multiverse framework extends beyond preregistration or traditional sensitivity analyses, which typically examine only a limited subset of alternatives. It formalizes the systematic exploration and communication of analytical flexibility, providing a principled way to identify decisions that disproportionately influence results as well as those that yield stable, convergent findings. This is particularly relevant in neuroimaging and network neuroscience, where analytical pipelines involve numerous interdependent steps (such as motion correction, denoising, parcellation, connectivity estimation, and graph-theoretic modeling) each offering multiple defensible implementations. Explicitly mapping this decision space allows researchers to detect effects that generalize across analytical conditions and to transparently document how their conclusions depend on specific analytical choices.

**Fig. 1.**
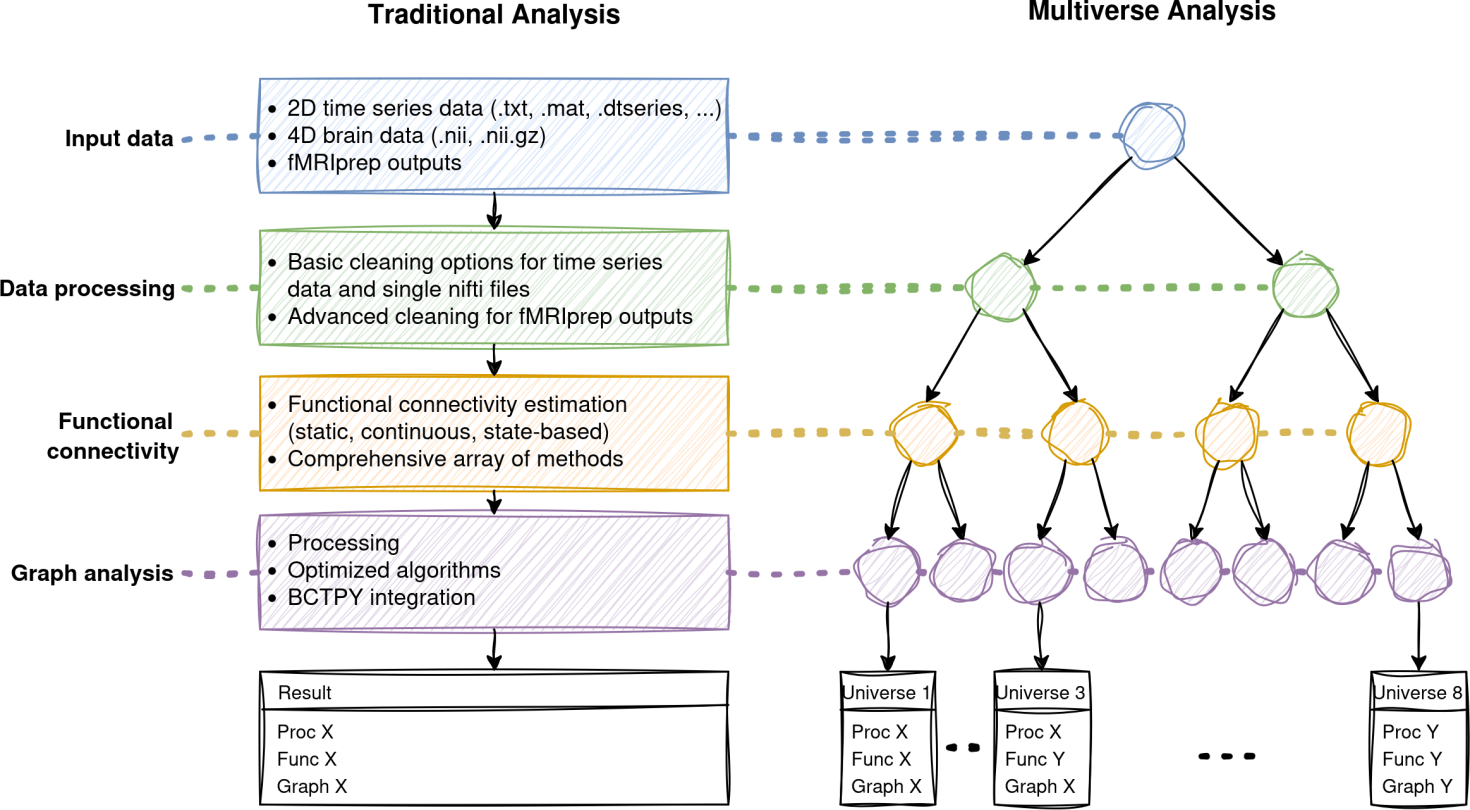
Features of the Comet Toolbox. Comet incorporates a broad set of functionalities spanning input handling, data processing, functional and dynamic connectivity estimation, and graph-theoretic analysis. On the left, a traditional single-pipeline analysis is illustrated, where a single result is produced from a fixed sequence of choices, with the available toolbox features listed in each box. On the right, the multiverse analysis framework is shown, illustrating possible combinations of defensible analytical decisions across the pipeline. Each branching point represents a user-specified set of alternatives at a particular analysis stage. The toolbox supports multiple input formats and includes data cleaning and preprocessing options. Connectivity estimation covers static, continuous, and state-based approaches, with a broad range of algorithms available. The graph module includes tools for network processing (e.g., thresholding, binarisation), widely used graph-theoretic measures, and integration with the Python implementation of the Brain Connectivity Toolbox (BCTPY). In the result boxes, each entry reflects a unique combination of processing, connectivity, and graph-analysis choices, referred to as a “universe” in the multiverse framework. Users can also integrate their own custom methods at any step, ensuring full extensibility.

To address the challenges outlined above, we introduce Comet: a user-friendly open-source Python toolbox designed specifically for multiverse analysis in functional connectomics and network neuroscience (Fig. 1). Comet provides an explicit and extensible framework for defining, executing, and visualizing multiverse analyses across connectivity estimation and graph-theoretic workflows. It supports a wide range of static, continuous, and state-based methods for estimating functional connectivity, and allows users to construct, compare, and evaluate complete analysis pipelines. Its modular design further enables the integration of user-defined methods at any point in the workflow, making it flexible and adaptable to a broad range of analytical configurations and research questions. The toolbox is accessible both through a graphical user interface (GUI) and via Python scripting, allowing seamless integration into automated and reproducible workflows. In addition, Comet includes extensive learning resources, including example pipelines, tutorials, and visual reporting tools, to support both novice and experienced users. Development of the toolbox is ongoing, ensuring that it continues to evolve in step with methodological advances in the field.

Comet is publicly available at https://github.com/mibur1/comet. In the sections that follow, we describe its architecture and core functionalities, present illustrative applications to multiverse analyses, and outline future developments aimed at broadening its utility for the neuroimaging community.

## 2 Methods

**Fig. 2.**
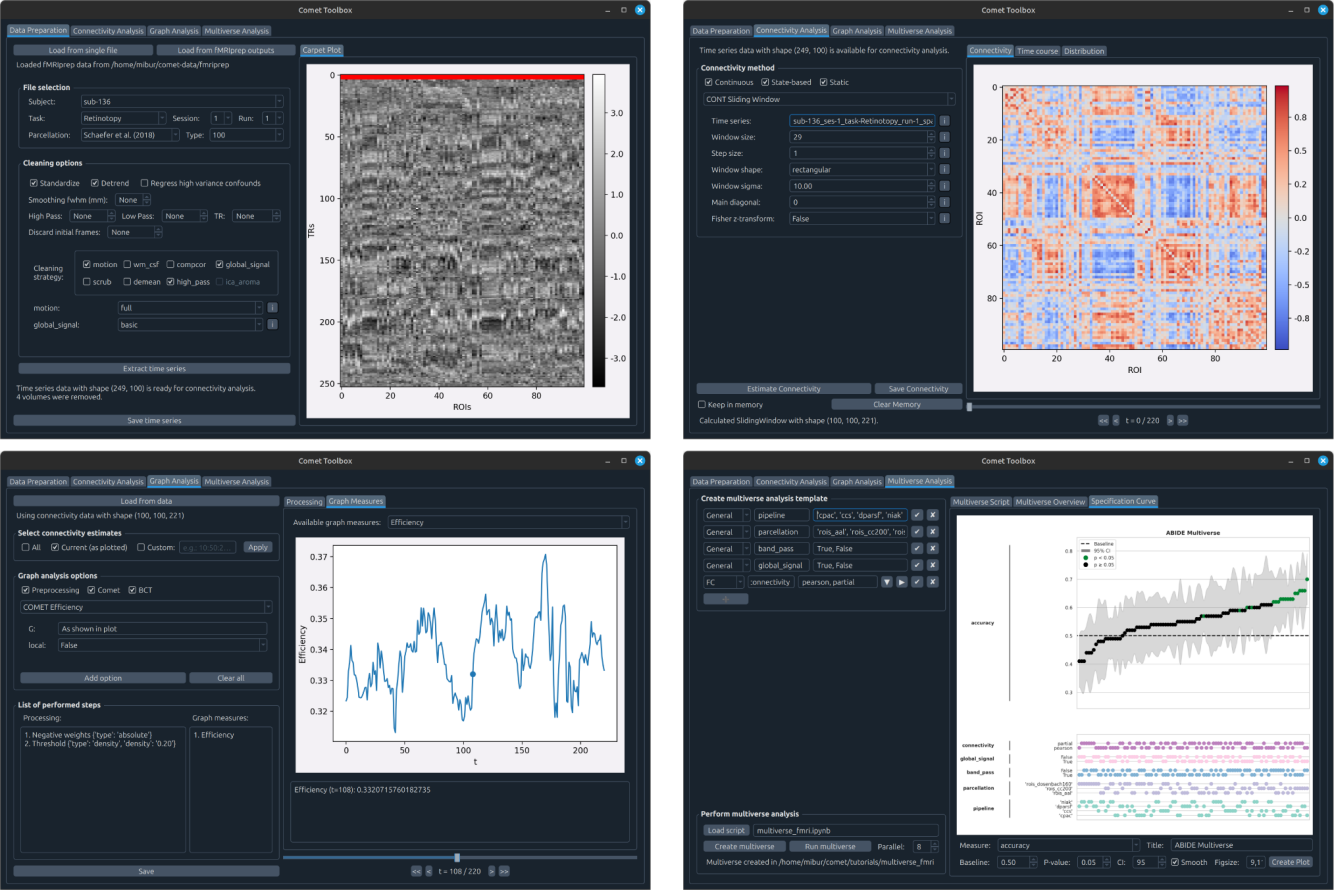
Graphical user interface. Comet offers a graphical user interface for most of its features, including data handling and processing (top left), functional connectivity estimation (top right), graph analysis (bottom left), and multiverse analysis (bottom right). All features can be used individually or in combination, allowing for a flexible usage.

The main contribution of this work is an open-source Python toolbox for multiverse analysis, with a focus on network neuroscience. The toolbox provides both a graphical user interface (Fig. 2) and a flexible scripting API (Figs. 5 and 6) to accommodate diverse workflows. A brief overview of the included features and methods will be provided in the following subsections.

### 2.1 Functional connectivity

The estimation of functional connectivity can be broadly divided into three distinct classes of methods, based on how temporal variations are addressed: Static functional connectivity, continuously varying dynamic functional connectivity, and state-based dynamic functional connectivity.

*Static functional connectivity* methods assume that connectivity remains constant throughout the duration of the recording, yielding a single overall connectivity estimate (Van Den Heuvel & Pol, 2010). In contrast, dynamic approaches aim to capture temporal fluctuations in connectivity (Iraji et al., 2021).

*State-based dynamic functional connectivity* methods characterize brain connectivity as a sequence of transitions between a finite set of discrete states, each assumed to exhibit quasi-stationary connectivity patterns. Transitions between these states reflect dynamic, time-dependent reconfigurations of the underlying functional brain network.

*Continuously varying dynamic functional connectivity* methods estimate connectivity at each time point (or a specified set of time points) and can further be divided into two sub-classes. Amplitude-based methods operate directly in the time domain of the signal, deriving connectivity estimates from the amplitude of BOLD time series data. Alternatively, time-frequency-based methods incorporate both frequency and phase information, revealing insights into temporal synchronization and rhythmic coordination between brain regions (see, for example, Lurie et al. (2020)).

Comet currently includes 18 different methods spanning these categories as summarized in Table 1. The methodological diversity exists not only between different approaches but also within individual methods, for example, in the choice of hyper-parameters such as the sliding window size or the number of connectivity states making them an ideal subject for multiverse analysis. All connectivity methods operate on 2D time series data ($n_{\text{time points}}\times n_{\text{brain regions}}$ or $n_{\text{time points}}\times n_{\text{voxels}}$ ) and return the full connectivity estimates, either as continuously varying time-resolved matrices or as state labels.

**Table 1.** Functional connectivity methods included in the Comet toolbox.^a^

Type	Method	Domain	Reference
Static	Pearson correlation	Amplitude	–
	Partial correlation	Amplitude	–
	Mutual information	Amplitude	–
Continuous	(Tapered) Sliding window correlation	Amplitude	–
	Flexible least squares	Amplitude	Liao et al. (2014)
	Multiplication of temporal derivatives	Amplitude	Shine et al. (2015)
	Spatial distance	Amplitude	Thompson et al. (2017)
	Jackknife correlation	Amplitude	Richter et al. (2015)
	Edge-centric connectivity	Amplitude	Faskowitz et al. (2020)
	Dynamic conditional correlation	Amplitude	Lindquist et al. (2014)
	Phase synchronization	Time-freq	Honari et al. (2021)
	Wavelet coherence	Time-freq	Billings et al. (2021)
	Leading eigenvector dynamics	Time-freq	Cabral et al. (2017)
State-based	Sliding window clustering	Amplitude	Allen et al. (2014)
	Co-activation patterns	Amplitude	X. Liu and Duyn (2013)
	Discrete hidden Markov model	Amplitude	Vidaurre et al. (2017)
	Continuous hidden Markov model	Amplitude	Ou et al. (2015)
	Windowless	Amplitude	Yaesoubi et al. (2018)

a For a full list of parameters and examples, refer to the toolbox documentation.

To accommodate diverse analytical needs, Comet also includes optional clustering-based state analysis for continuously varying dFC measures. This enables users to obtain standard state-based summary measures such as dwell times, fractional occupancy, or transition probabilities for all included connectivity methods.

### 2.2 Graph analysis

**Fig. 3.**
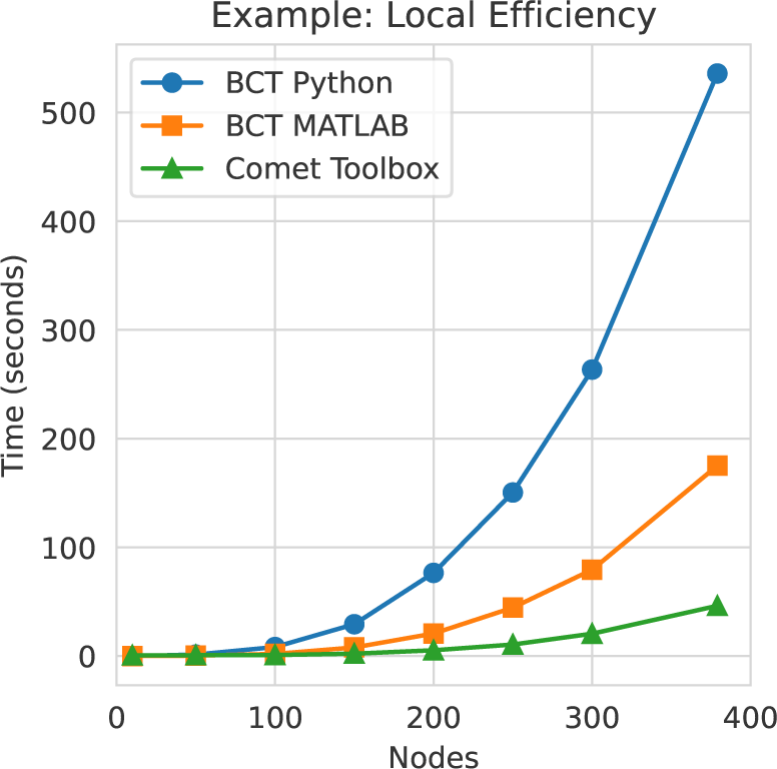
Toolbox performance. Implementations of computationally heavy graph measures included in Comet compile to native machine code at run time, which significantly improves performance. For example, on a laptop equipped with an AMD Ryzen 7 PRO 7840U, the calculation of local efficiency for a weighted brain network with 379 nodes at 50% density shows improved calculation speed compared to the Python version (>10x) and MATLAB version (>3x) of the Brain Connectivity Toolbox (BCT).

Comet offers a range of graph-analysis techniques for estimating network topology within a multiverse analysis framework. To facilitate this, it provides wrapper functions for most of the algorithms available in the Python implementation of the Brain Connectivity Toolbox (Rubinov & Sporns, 2010). Further, for performance-related reasons, Comet also offers optimized implementations for the following computationally heavy algorithms: global efficiency, local efficiency, shortest average path length, and the matching index. These implementations are compiled to machine code at run time using the Numba library (Lam et al., 2015). This is crucial for multiverse analysis, as while computation time for the entire multiverse can simply be parallelized over the universes, the computation time for the individual graph measures within each universe scales steeply with increasing network size. An example of the improved performance is shown for the calculation of local efficiency. In a network containing 379 nodes, the computation is between 3 and 10 times faster than in previous implementations of the method (Fig. 3). A complete list of the currently included methods can be found in the documentation of the toolbox.

### 2.3 Multiverse analysis

Comet implements a flexible and modular framework for conducting multiverse analyses. Users specify the possible alternatives for each analytical decision, thereby defining the forking paths within their analysis pipeline. The toolbox then automatically generates analysis scripts for all possible combinations of these decision options (or for a selected subset based on pruning rules). Each unique combination of decisions constitutes a separate universe within the multiverse framework, representing one defensible analytical pathway from data processing and analysis to result. Universes can then be evaluated and their outcomes are aggregated to assess the robustness of findings. This design allows users to easily inspect individual Python scripts and provides the flexibility to execute them either sequentially or in parallel, depending on the available computational resources. A brief illustration of the multiverse workflow is provided in Figure 6 and will be discussed in more detail in Section 3.2.

## 3 Usage

**Fig. 4.**
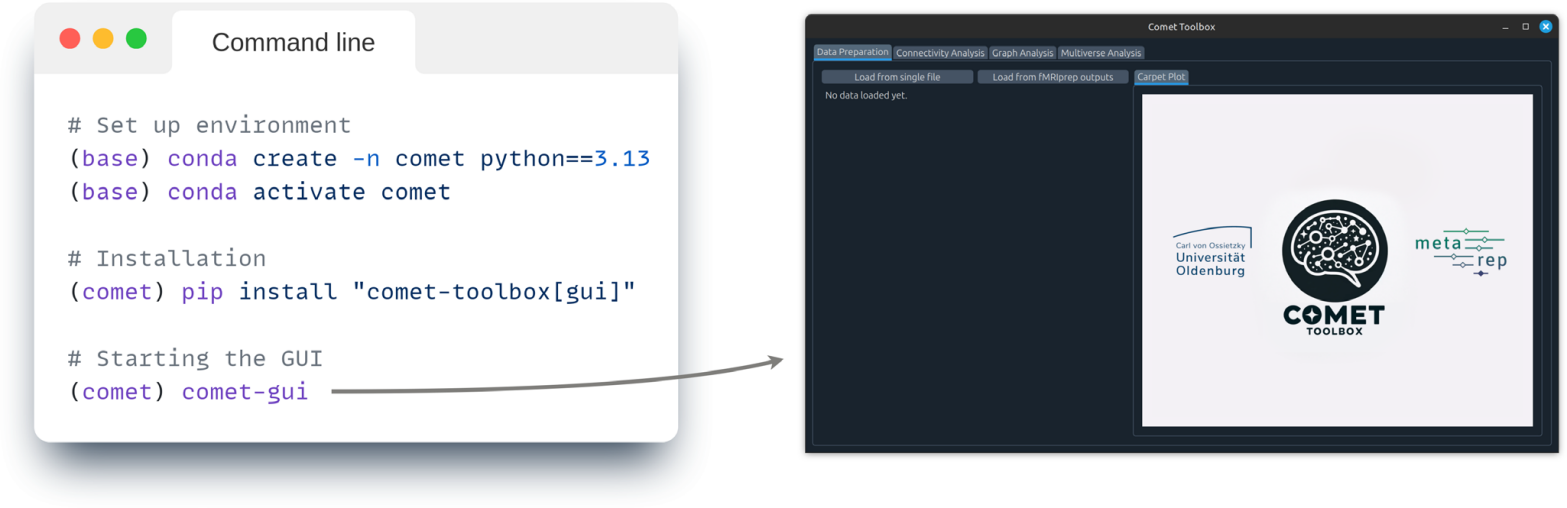
Installation and usage. Comet can be installed via the Python Package Index (PyPI) using pip install. Due to the extensive set of dependencies, we strongly recommend installing Comet within a dedicated environment. Once installed, the graphical user interface (GUI) can be launched by using the comet-gui command.

The Comet toolbox can be installed via the Python Package Index (PyPI). As this will automatically install or update required packages, we strongly recommend users to set up a dedicated environment (e.g., venv^1^ or conda^2^) to avoid potential version conflicts (Fig. 4).

A key design goal of the toolbox is to support flexible and modular use. Researchers do not need to run their entire analysis pipeline within Comet. Instead, the toolbox can be used as a standalone tool to estimate dynamic functional connectivity or to conduct multiverse analyses. This makes it suitable for a wide range of study designs and research questions. At the time of this publication, Comet includes five main modules, which are described in detail in the documentation:

comet.connectivity (Dynamic) functional connectivity methods,
comet.graph Graph analysis functions,
comet.multiverse Multiverse analysis functions,
comet.utils Data loading and processing utilities,
comet.cifti CIFTI parcellation and file handling.

This modular design allows users to integrate individual components of the Comet toolbox, such as specific functional connectivity estimation methods, into their own analysis pipelines with minimal changes. For instance, users can apply Comet’s connectivity methods and then export the results for further processing in other environments such as MATLAB. Similarly, the multiverse analysis module operates independently of the specific methods implemented in Comet, meaning it can be flexibly applied to any kind of multiverse analysis pipeline, including those built with user-defined or external functions.

To accommodate different user needs and workflows, Comet provides both a graphical user interface (GUI) and a standard scripting interface. While scripting offers greater flexibility and automation, the GUI supports all core functionalities and is particularly useful for exploratory analysis and teaching. After installation, the GUI can be launched directly from the command prompt/terminal (Fig. 4).

### 3.1 Graphical user interface

The graphical user interface currently includes four tabs: Data Preparation, Connectivity Analysis, Graph Analysis, and Multiverse Analysis (as shown in Fig. 2).

1. In the *Data Preparation* tab, users can import the desired data. This includes time series data of shape $\left(N_{\text{time points}}\times N_{\text{brain regions / voxels}}\right)$ in all popular formats (.txt, .mat, .npy,…), standard fMRI images (.nii,.dtseries), or an entire fMRIprep processed dataset. Depending on the input type, the interface dynamically offers appropriate preprocessing options such as parcellation, temporal filtering, and denoising. The resulting time series data can then either be saved to disk or passed forward for connectivity analysis.
2. In the *Connectivity Analysis* tab, users can select any of the included functional connectivity methods, and apply them to the data as prepared in the previous tab. The *Keep in memory* checkbox allows users to keep the calculated dFC estimates in working memory to enable quick switching and comparison between different methods. For computationally expensive dFC methods, a progress bar will be displayed in the terminal from which the GUI was invoked. Once computed, the connectivity matrices are automatically visualized in the right-hand panel, and users can explore region-to-region connectivity time courses, inspect summary statistics, or export results for external use.
3. In the *Graph Analysis* tab, users can compute a range of graph measures from connectivity matrices. The GUI accepts input in the form of either static connectivity matrices of shape $N_{\text{brain regions}}\times N_{\text{brain regions}}$ or dynamic connectivity data with an additional temporal dimension $N_{\text{brain regions}}\times N_{\text{brain regions}}\times N_{\text{time points / states}}$ . These can be uploaded directly or selected from previously computed results. The interface supports the calculation of commonly used graph metrics such as global efficiency, modularity, degree centrality, clustering coefficient, and community detection.
4. In the *Multiverse Analysis* tab, users can define and evaluate multiverse analyses. This involves specifying a set of decisions, each with multiple options, which are inserted as placeholders into a template script. The template represents the user’s analysis pipeline and can either be written directly in the GUI or exported and edited externally. Once the analysis script is complete and includes all placeholders, it can be reloaded into the GUI for execution and result visualization. Because this process requires users to write parts of the analysis code themselves, it closely resembles the scripting-based workflow shown in Figure 6. While fully usable, users might generally prefer to perform multiverse analyses with a pure script-based approach as this offers enhanced usability through features of the programming editors such as syntax highlighting, better error handling, or integrated documentation.

### 3.2 Scripting API

**Fig. 5.**
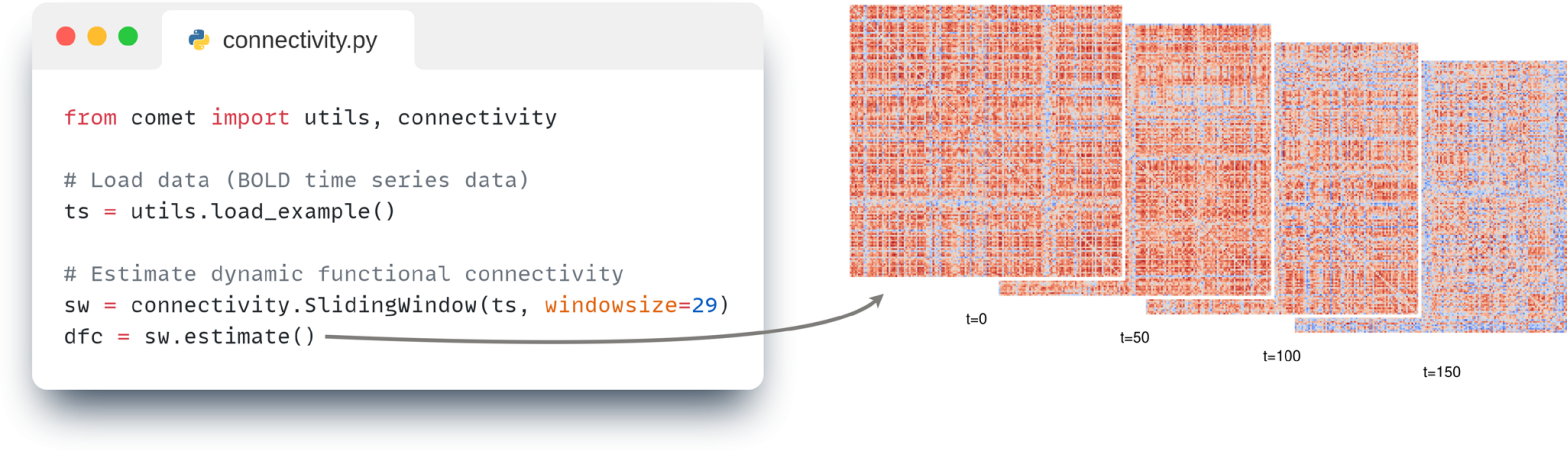
Functional connectivity. Left: Code for estimating (dynamic) functional connectivity. All methods are provided as individual classes, functional connectivity can be estimated through the .estimate() method. Right: The resulting sliding window connectivity estimates at four exemplary time points.

If more complex analyses are required or a scripting approach is preferred, Comet provides a standard scripting interface that allows direct use of individual modules. For example, as shown in Figure 5, a dynamic functional connectivity method can be used by first importing and instantiating the corresponding class (e.g., SlidingWindow()) from the connectivity module, and then calling its estimate() method. In this example, example time series data are loaded from the utils module. A complete list of available classes, methods, and parameters is provided in the online documentation linked at https://github.com/mibur1/comet.

Multiverse analyses can be implemented programmatically via the multiverse module in standard Python scripts or in Jupyter Notebooks. This approach allows full control over the forking paths, analysis templates, and optional configurations. Forking paths (i.e., decision points with multiple possible options) are defined in a Python dictionary. Each combination of options corresponds to a unique analysis pipeline, referred to as a *universe*.

**Fig. 6.**
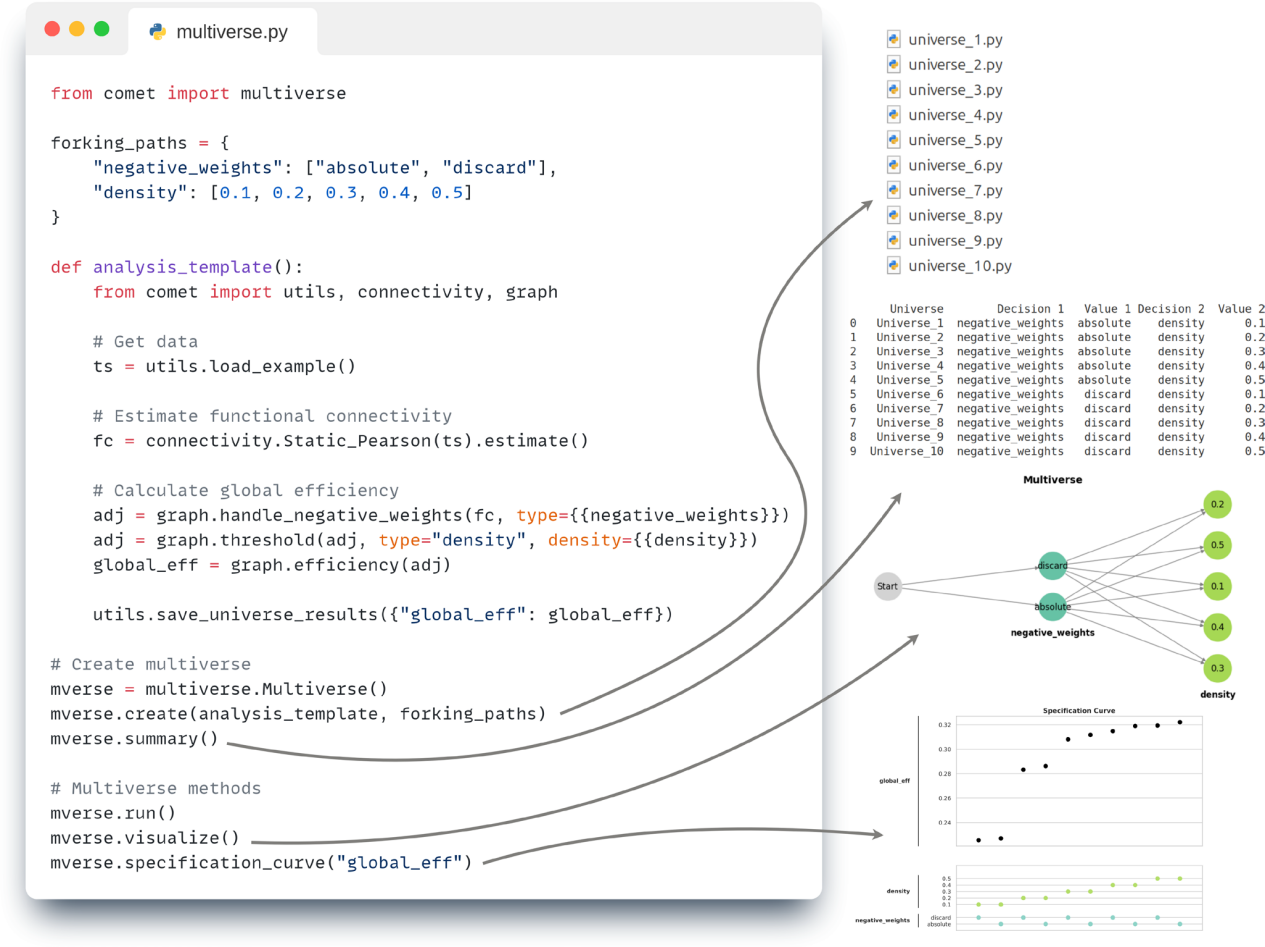
Multiverse analysis workflow in the Comet toolbox. Left: A minimal example of a multiverse analysis script is shown. Forking paths are defined as a dictionary of decision points and their options (e.g., how to handle negative_weights and which density thresholds to apply). These are inserted as placeholders (e.g., {{density}}) into a template function, which defines the analysis logic (in this case, estimating global efficiency from static functional connectivity). The multiverse is then created using the .create() method, and evaluated using the .run() method. Right: The toolbox automatically generates Python scripts (e.g., universe_1.py, universe_2.py,…) for each valid combination of options, referred to as a *universe*. Each script represents a fully defined analysis pipeline and can be viewed and analyzed individually. A table summarizes the generated universes, with each row showing the specific options chosen for each decision. The multiverse structure can be visualized as a decision graph, where each branching point represents a forking path. Finally, the results across all universes are summarized using a specification curve, which allow users to identify how different analytical choices affect the outcome (in this case global efficiency).

An example using two forking paths is shown in Figure 6. The first forking path (negative_weights) defines two options for handling negative values in a connectivity matrix. The second forking path (density) specifies five possible thresholds for graph construction. These options are represented in the analysis script by placeholders written in double curly brackets, such as {{density}}. During multiverse generation, these placeholders are automatically replaced by each of the defined options, allowing the toolbox to generate a separate analysis script for all valid combinations of decisions.

Once the template and forking paths are defined (in this simple example a pipeline for estimating global efficiency from static functional connectivity), the multiverse is created using the create() method, which generates individual Python scripts for each universe (e.g., universe_1.py, universe_2.py, etc.). These scripts can be executed directly using the .run() method, either sequentially or in parallel. The multiverse can be created, analyzed, and visualized, with the most important built-in functions being:

multiverse.Multiverse() Initializes the multiverse object,
.create() Generates universes and corresponding scripts,
.summary() Provides an overview of decisions and universes,
.visualize() Plots the structure of the multiverse,
.run() Evaluates the universes (in full or in part),
.specification_curve() Visualizes variability across universes,
.get_results() Get all (or parts) of the results,

In the example shown, the two forking paths with 2×5 options yield 10 unique universes. Each universe script contains a fully specified analysis pipeline corresponding to one combination of decisions. The multiverse structure is shown as a branching graph, and the results are summarized in a specification curve. More advanced features, such as defining invalid decision combinations or specifying decision orders, are supported via an optional configuration dictionary. Detailed tutorials are available in the documentation.

## 4 Analysis Example

We showcase the multiverse analysis workflow through a simple example using resting-state fMRI data from the Autism Brain Imaging Data Exchange (ABIDE) (Craddock et al., 2013). The goal of this analysis is to predict an autism diagnosis from static functional connectivity estimates. Data from 871 participants (403 autism, 468 controls) were included using the publicly available preprocessed connectomes dataset (http://preprocessed-connectomes-project.org/abide/index.html). The example presented here closely parallels the classification multiverse conducted by Dafflon et al. (2022), with the main difference being that we considered a slightly reduced decision space for the connectivity measures and parcellation, but added a decision point for the regularization strength of the classifier to also cover the statistical model within the multiverse. The final multiverse contains 192 universes, incorporating critical analytical decisions for data preprocessing, parcellation, connectivity estimation, and the classification model. A summary of these forking paths is provided in Table 2. The accompanying Python Notebook is included in the toolbox documentation.

**Table 2.** Decisions and options for the example analysis.^a^

Decision	Option
Processing pipeline	CPAC
	CCS
	DPARSF
	NIAK
Parcellation	AAL
	CC 200
	Dosenbach 160
Global signal regression	True
	False
Bandpass filter	True
	False
Connectivity measure	Pearson correlation
	Partial correlation
Regularization strength	0.25
	1.0

a The multiverse consists of 192 unique combinations of decisions (4×3×2×2×2×2), referred to as universes. For each universe, a logistic regression classifier was trained to predict autism diagnoses based on features extracted from the corresponding functional connectivity matrices. Results of these classification analyses are summarized in Figure 7.

In detail, the multiverse analysis included data from four preprocessing pipelines available in the ABIDE dataset: the Configurable Pipeline for the Analysis of Connectomes (CPAC), the Connectome Computation System (CCS), the Data Processing Assistant for Resting-State fMRI (DPARSF), and the NeuroImaging Analysis Kit (NIAK). Furthermore, we included data parcellated with three different brain atlases: the Automated Anatomical Labeling atlas (AAL), the Cameron and Craddock 200 functional parcellation (CC 200), and the Dosenbach 160 atlas. Each preprocessing-parcellation combination was further varied by including or excluding bandpass filtering and global signal regression (for more details on the processing pipelines, please visit the official website at http://preprocessed-connectomes-project.org/abide/Pipelines.html). Static functional connectivity was then estimated using either Pearson or partial correlation. The resulting functional connectivity matrices were vectorized and used as input features for a logistic regression classifier with L2 regularization. Classification performance was assessed using stratified 5-fold cross-validation. The results were visualized using a specification curve (Fig. 7), which arranges all pipeline configurations by their classification accuracy in ascending order. This provides a comprehensive view of how analytical choices affect model performance.

**Fig. 7.**
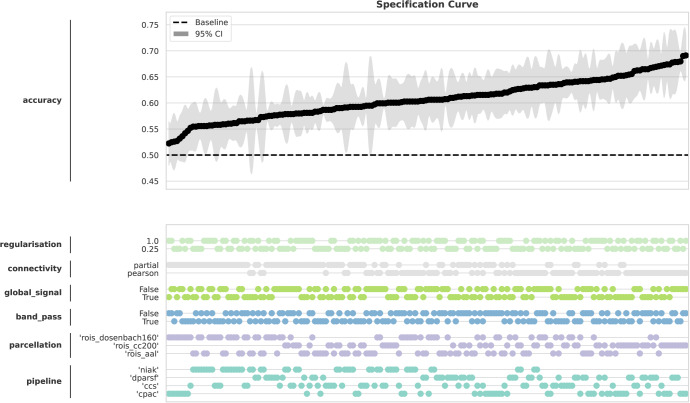
Specification curve for the example analysis. The classification accuracies for the individual universes are displayed in ascending order. The upper plot displays the classification accuracies with their 95% confidence intervals resulting from 5-fold cross-validation. The bottom plot displays the individual decisions chosen in the analysis pipeline leading to the corresponding classification accuracy above.

Notably, accuracies varied widely across universes, with some pipelines performing close to chance while others achieved accuracies in the range of 70%. This highlights how analytical choices may critically affect outcomes in predictive modeling of clinical diagnoses. We emphasise that the purpose here is to demonstrate the functionality of the toolbox rather than to justify specific analytical decisions or interpret the resulting outcomes. Moreover, the classification example represents a typical yet simplified analysis pipeline for fMRI biomarker research. We further emphasize that the multiverse framework implemented in the Comet toolbox is entirely agnostic to the type of analytical workflow and can flexibly accommodate pipelines with any set of decision points. Further examples, including non-neuroimaging applications, are provided in the online documentation.

## 5 Discussion

The present work introduced the Comet toolbox, a purpose-built framework for conducting multiverse analyses within network neuroscience. We believe the toolbox addresses a critical need in a rapidly growing field of research, where multiverse analysis is increasingly used to assess the robustness of findings in fMRI studies. For example, Luppi et al. (2024) conducted a large-scale multiverse analysis of processing pipelines to promote more consistent practices in functional connectomics, while Demidenko et al. (2024) applied multiverse analysis to explore the influence of preprocessing decisions in the context of the monetary incentive delay task. In parallel, more conceptual contributions such as Lefort-Besnard et al. (2025) have framed multiverse analysis as a form of same data meta-analysis and proposed integration strategies for evaluating analytical variability, although these efforts have so far been limited to test statistic maps from activation studies.

At the same time, broader methodological needs in network neuroscience remain unresolved. For example, Fekonja et al. (2025) recently outlined nine key roadblocks that hinder clinical adoption of network-based methods, including difficulties in constructing accurate and interpretable networks, the computational complexity of dynamic connectivity analysis, and the lack of accessible, user-friendly tools suitable for clinical workflows. Comet aims to address several of these barriers by providing (i) a systematic framework for exploring analytical flexibility, (ii) integrated modules for functional connectivity and graph-theoretic analysis, and (iii) a dual interface (graphical and script-based) designed to be accessible to users regardless of their technical background.

### 5.1 Comparison to existing software

Comet aims to address a specific methodological gap in network neuroscience by combining dynamic functional connectivity (dFC) estimation, graph-theoretical analysis, and multiverse workflows within a single, unified platform. Although various toolboxes are available for individual aspects of this workflow, few provide an integrated framework that supports both methodological diversity and systematic multiverse analysis. While not exhaustive, the following subsections outline related toolboxes and approaches to situate Comet within the current software landscape.

Many existing packages implement one or a few specific dFC methods, often developed alongside individual publications. These include, for example, the timecorr package for dynamic higher-order correlation (Owen et al., 2021), the Dynamic Correlation toolbox for GARCH-based models (Lindquist et al., 2014), the TbCAPs toolbox for co-activation pattern analysis (Bolton et al., 2020), or implementations of Leading Eigenvector Dynamics (Cabral et al., 2017; Olsen et al., 2022). While these tools provide valuable methodological contributions, their individual scope, and in some cases their tight integration with publication specific workflows, can limit their reusability and accessibility.

Some Python and MATLAB-based toolboxes offer broader functionality for dFC analysis. For example, the Python toolboxes teneto (Thompson et al., 2017) and pyDFC (Torabi et al., 2024) support multiple dFC methods and allow for modular analysis pipelines. However, neither is focused on complex multiverse workflows, and both rely on internal data formats that may require additional steps to integrate with widely used neuroimaging toolchains such as the nilearn Python package. In MATLAB, toolboxes like DynamicBC (Liao et al., 2014), CONN (Nieto-Castanon, 2020), GIFT (Iraji et al., 2021), or NaDyNet (Yang et al., 2025) include support for dFC estimation and often also state-based clustering, but do not provide explicit tools for multiverse analysis or analytical comparison across parameter spaces. In contrast, Comet is designed to support structured exploration of analytical variation and integrates both various approaches of dFC estimation and graph-based analyses in a way that is directly compatible with multiverse workflows. Notably, Alteriis et al. (2025) proposed the Dysco framework as an alternative strategy for reducing analytical uncertainty by combining multiple dFC methods into a single integrated approach. While this differs from the multiverse framework used in Comet, it reflects a shared aim of improving robustness by addressing uncertainty across methodological choices.

Other packages focus on full pipeline integration, from preprocessing to graph-theoretic analysis. Examples include GRETNA (Wang et al., 2015), which supports static and dynamic connectivity analysis with graph metrics, and ACTION (Fang et al., 2025), which provides a GUI-based pipeline for static functional connectivity and machine-learning models. While these toolboxes offer end-to-end functionality, they do not incorporate multiverse analysis features, and typically support only a limited selection of connectivity and graph-theoretic methods. In contrast, Comet explicitly supports fully flexible, user-defined decision structures and systematic multiverse analysis.

Finally, outside the domain of dFC and network neuroscience, a number of general-purpose multiverse analysis tools have emerged. These include Boba, multiverse, and specr (Y. Liu et al., 2020; Masur & Scharkow, 2020; Sarma et al., 2023), which were designed for general statistical modeling in the behavioral and social sciences and, except for Boba, are implemented in the R programming language (R Core Team, 2025). Other packages focus on specific experimental domains, such as fear conditioning (Lonsdorf et al., 2022) or the visualization of multiverse results (Sarma et al., 2024). While these tools offer important contributions to the broader discussion on analytical flexibility, they are typically not tailored to the complexity of neuroimaging workflows. In contrast, Comet was developed specifically to address the needs of fMRI and network neuroscience research.

In summary, while existing toolboxes offer powerful features for specific parts of the analysis pipeline, none provide the methodological diversity, extensibility, and integration needed to support multiverse analyses in the context of dFC and network neuroscience. Comet is designed to bridge this gap by combining established and novel methods for dFC, flexible graph analysis tools, and a dedicated multiverse workflow, all within a single, accessible framework.

### 5.2 Challenges and interpretational considerations

The general framework of multiverse analysis provides a principled way to make analytical flexibility explicit and to address the analytical variability inherent in neuroimaging results. However, it comes along with several ongoing challenges. The first challenge lies in defining and analysing the often large analytical decision space. As additional decision nodes are added, the number of possible analytical pathways grows rapidly, potentially leading to a combinatorial explosion in the multiverse. This introduces the risk that plausible effects become obscured when insufficiently justified analytical options are included.

A second challenge concerns the interpretation of multiverse results. Two complementary perspectives have emerged in the methodological literature. One emphasizes integration across the decision space, seeking to summarize or aggregate results to identify effects that remain consistent across many defensible specifications (e.g., Cantone & Tomaselli, 2024). The other focuses on highlighting variability, examining how results differ across specifications, and identifying decision points that systematically influence outcomes (e.g., Bowring et al., 2022).

Together, these considerations demonstrate that both the construction and interpretation of multiverse analyses involve conceptual choices of their own. Rather than endorsing a single viewpoint, the multiverse framework, as implemented in the Comet toolbox, is designed to accommodate diverse analytical philosophies and to provide a structured starting point for examining how methodological uncertainty shapes scientific results.

### 5.3 Future directions

Comet is an actively developed toolbox and will continue to evolve over time. Looking ahead, several possible directions for expansion may further enhance the utility and scope of the toolbox. Beyond its current primary use in assessing the robustness of results across analytical choices (e.g., via specification curve analysis; Simonsohn et al. (2020)), future developments will explore additional conceptual perspectives. For instance, multiverse analysis has recently been proposed as a tool for identifying covariates that substantially influence outcomes (Bowring et al., 2022), or as a framework for supporting abductive reasoning and inferential decision-making in complex analyses (Engzell & Mood, 2023). Related efforts in the fMRI domain are beginning to investigate approaches for statistical inference and multiverse integration (Cantone & Tomaselli, 2024; Girardi et al., 2024; Lefort-Besnard et al., 2025; Ozenne et al., 2025), and could inform future methodological modules in Comet. Techniques such as active learning have also been suggested to optimize multiverse exploration when a full combinatorial search is infeasible (Dafflon et al., 2022). While these developments remain at the research frontier, they highlight promising avenues for extending functionality. Future versions of the toolbox might also incorporate temporal network measures that go beyond static snapshot topology, as described by Thompson et al. (2017). Additionally, tools for studying inter-individual differences from a psychometric perspective are not yet integrated, despite their relevance for personalised network neuroscience (Forkel et al., 2022).

Taken together, we see Comet not as a static software package but as a flexible and extensible platform that can evolve with the methodological needs of the neuroimaging community. In this sense, multiverse analysis becomes a starting point for refining hypotheses, deflating the decision space, and contributing to theoretical unification.

### 5.4 Conclusion

The Comet toolbox provides a unified yet flexible platform for connectivity and network analysis, enabling researchers to systematically explore how methodological choices influence study outcomes. It integrates a broad range of dFC methods alongside graph-theoretical tools. Its multiverse analysis framework addresses growing concerns about robustness and reproducibility in neuroimaging by facilitating structured comparisons across diverse analytical pipelines. The resulting outputs are designed to support a deeper understanding of how analytical variability affects research findings.

Comet is accessible through both a graphical user interface and a simple scripting API, making it suitable for users with varying levels of programming expertise. By supporting both conventional and multiverse-style workflows, the toolbox serves as a valuable resource for enhancing transparency, flexibility, robustness, and reproducibility in neuroimaging research. The toolbox and documentation are available at: https://github.com/mibur1/comet.

## Ethics

No data were collected as part of this study. The ABIDE data used in the analysis example are fully de-identified and publicly available; therefore, no additional ethical approval was required for their use.

## Data Availability

The data, code, and documentation for the toolbox are publicly available at https://github.com/mibur1/comet. The documentation website provides comprehensive guidance for installation and usage, offering a broad range of tutorials and API information.

## Footnotes

1
https://docs.python.org/3/library/venv.html

2
https://conda-forge.org/download/
